# Editorial: Optimizing player health, recovery, and performance in basketball, volume II

**DOI:** 10.3389/fpsyg.2025.1577701

**Published:** 2025-03-06

**Authors:** Aaron T. Scanlan, Daniele Conte, Davide Ferioli

**Affiliations:** ^1^School of Health, Medical and Applied Sciences, Central Queensland University, Rockhampton, QLD, Australia; ^2^Department of Movement, Human and Health Sciences, University of Rome “Foro Italico”, Rome, Italy; ^3^Department of Biomedical Sciences, Dental Sciences, and Morpho-Functional Imaging, University of Messina, Messina, Italy

**Keywords:** training, monitoring, testing, technical, fatigue, load, wheelchair, referee

## Introduction

**Figure 1:**
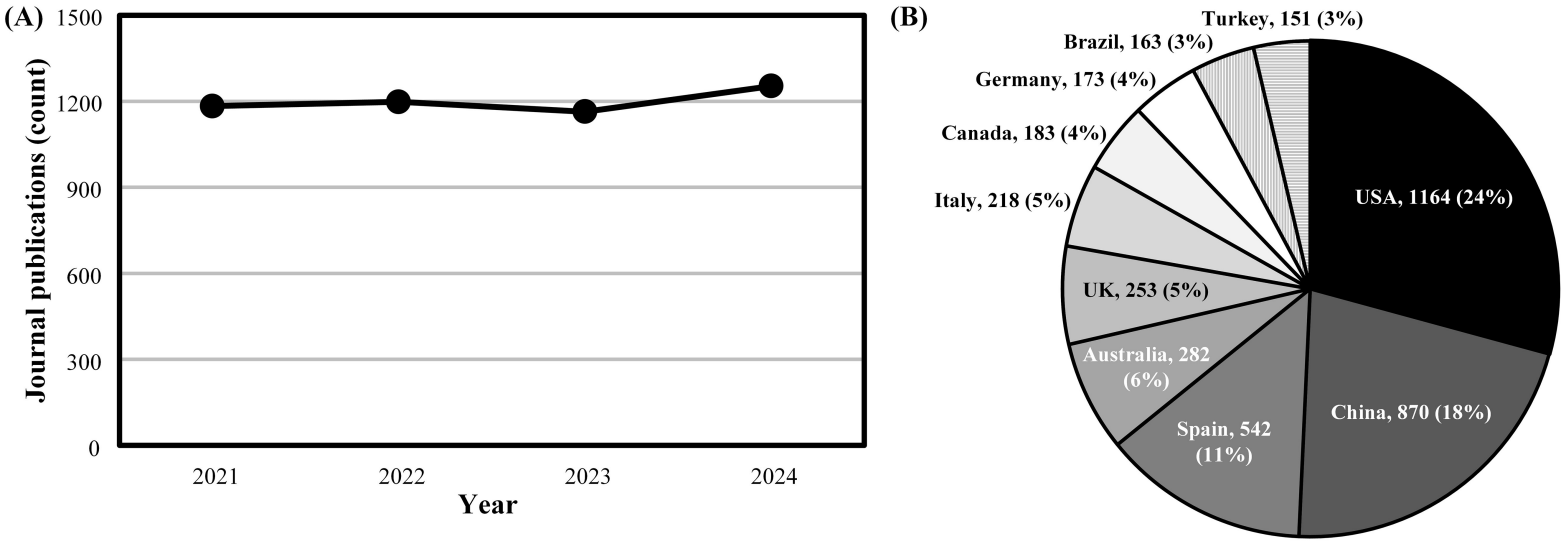
Scopus search results between 2021 and 2024 showing **(A)** yearly trends in total basketball journal publications and **(B)** the proportion of publications according to the country of the leading author. The term “basketball” was searched within the “Article title, Abstract, Keywords” field, with “Journal” selected as source type and “Article in Press” excluded on 14 February 2025. Total publications alongside the proportion (%) of these publications relative to all basketball journal publications are shown in **(B)**.

This second Research Topic on “*Optimizing player health, recovery, and performance in basketball*” extends upon the first Research Topic in this area we edited (Ferioli et al., 2022). In this regard, we noted the upward trajectory in journal publications focused on basketball between 2002 and 2021 previously (Ferioli et al., 2022), with outputs remaining consistently strong since this time (Figure 1A). This sustained output in basketball research might be attributed to the high participation rate and interest for the sport on a global scale (Hulteen et al., 2017), with basketball journal publications being authored by researchers from a wide dispersion of countries (Figure 1B). In support of this global attention for basketball research, data acquired from SciVal (retrieved 14 February 2025) indicate ~25% of basketball publications involve international collaborations and authors from >100 countries cite basketball publications on average each year (between 2021 and 2024). Indeed, the research published in this Research Topic involves authors from nine countries, directly showcasing the internationalization of evidence being generated in basketball.

## New insights provided in this Research Topic

The growth in basketball research across recent years has created more opportunities for researchers to synthesize evidence in areas of interest. Systematic reviews present a means to collate evidence on a particular topic to better inform decision-making processes among policy-makers, practitioners, researchers, and the public (Bangdiwala, 2024). In this regard, four systematic reviews, including three with meta-analyses, are published in this Research Topic, focusing on performance-related outcomes resulting from training interventions and fatigue. More precisely, Cao, Liu et al. and Cao, Wang et al. synthesized the literature across separate reviews to demonstrate the benefits of functional training for enhancing different physical performance outcomes across various basketball players (Cao, Liu et al.), and the positive outcomes of plyometric training on fitness and skill attributes specifically in female players (Cao, Wang et al.). Similarly, Zhou et al. showed plyometric training improves several physical performance outcomes among youth players, with specific insight provided according to age range, sex, and training protocols. Finally, Li et al. reported how some shooting performances were negatively impacted in response to varying extents of physical and mental fatigue among adolescent and adult players.

A notable element of this Research Topic concerns the inclusive nature of the participant samples examined across studies. In this regard, half of the published studies, including two original articles and four reviews, encompassed female participants, who have historically received far less research attention than males within the basketball literature (Paul et al., 2023; O'Grady et al., 2020). However, the sex breakdown in participants across studies included within the reviews published in this Research Topic further emphasize the deficiencies in research evidence specifically in female players across the topics examined. Consequently, there is a strong need for improved balance via effective research designs with female players in future work as advocated (Elliott-Sale et al., 2021; Smith et al., 2022). Moreover, other studies published in this Research Topic recruited participant samples that are not readily examined in the basketball literature. For instance, Yasuda et al. identified outcomes from various tactical strategies applied during screening scenarios in male, wheelchair basketball players during Paralympic competition. Likewise, Wang et al. focused on high-level basketball referees, showing that mood state was indirectly impacted by coping style with mediating roles of psychological resilience and frustration tolerance. It should be noted that although the title of this Research Topic was oriented toward players, referees are essential for the continuation of basketball competitions, with their contribution to games having the potential to directly impact player performance and health. In addition, Cabarkapa, Aleksic et al. showed that eccentric-based metrics derived from countermovement jump testing may be useful in detecting neuromuscular-related fatigue surrounding play in 3 × 3 basketball players. Indeed, we previously recommended (Ferioli et al., 2022) that more applied research should explore 3 × 3 basketball contexts given the rapid growth in this form of the sport (Sansone et al., 2023)—along with other innovative strategies that could positively impact practice in basketball settings.

Other studies in this Research Topic exploring innovative strategies yield outcomes that hold application in many important areas for basketball practitioners. In this regard, Ferioli et al. investigated a novel, game-specific basketball simulation protocol in male and female players with reliability and discriminative validity data provided to inform its utility for repeated testing occasions and selecting or benchmarking purposes. Using similar discriminatory analyses, Cabarkapa, Cabarkapa et al. showed various force-time metrics from countermovement jump testing do not differentiate between starting and non-starting, professional male players, limiting its utility in this way. Adopting a novel approach, Wellm et al. quantified the contact demands faced by professional, male players during games, showing they undergo regular physical contact during specific play scenarios with distinct profiles emerging for each playing position. Expanding beyond novel exploration of approaches to measure physical attributes, Hogan et al. demonstrated the importance of cognitive abilities (via the Athletic Intelligence Quotient) for performance among players in the National Basketball Association. Finally, Zhang et al. showed that a 10-week targeted unilateral compound training program reduced strength and power-related asymmetries in the lower limbs to enhance performance in these attributes. The evidence these studies provide regarding physical and psychological profiling, load measurement, and training approaches are highly relevant to end-users given they can inform practice in key areas in which research is used like load monitoring, strength and conditioning, mental training, and tactical strategies (Schwarz et al., 2021).

## Future research directions

When editing this Research Topic, some notable trends were identified regarding the areas examined and designs adopted across studies, which may help to inform future research pathways in basketball. First, many applied basketball studies, including those in this Research Topic, are descriptive, which is likely due to the accessibility of routinely collected data among basketball teams without the ability to manipulate approaches (e.g., training contents) in a controlled manner (Buchheit, 2019). Consequently, higher quality evidence stemming from well-planned interventional research is essential to identify the most efficacious and pragmatic approaches for specific contexts within basketball teams (Bishop, 2008). In this regard, several studies across various research groups are needed to identify the best intervention strategies for a particular area of interest (Bishop, 2008).

Second, no studies in this Research Topic gathered insights from end-users in practice. Input from basketball players, coaches, and other practitioners can help identify practical problems, which can assist in developing research questions that carry a stronger impact (Bishop, 2008). Likewise, perceptions from end-users can also help identify barriers and motivators for the uptake of new evidence to guide implementation research in real-world basketball settings (Bishop, 2008). In this way, more studies utilizing appropriate quantitative and qualitative methods to gather insights from end-users should be conducted to better ensure research outcomes effectively translate to practice (Abt et al., 2022). This type of approach is lacking in the wider basketball literature, with data from end-users scarcely reported in recent years and restricted to specific topics like recovery strategies (Pernigoni et al., 2022), flywheel training (Younes-Egaña et al., 2023), and injury prevention strategies (Bel et al., 2022). Moreover, data acquired from SciVal (retrieved 14 February 2024) indicate >70% of basketball publications involve academic-only collaborations (between 2021 and 2024), further emphasizing the need to involve end-users in the development, design, and conduct of basketball studies.

Third, we previously encouraged more interventional research exploring strategies to minimize injury risk, enhance return-to-play progression, and optimize recovery in basketball (Ferioli et al., 2022). No studies in this Research Topic examined injury prevention or rehabilitation strategies, which is surprising given the high volume of research historically focused on injuries in the basketball literature (Scanlan and Dalbo, 2019). Likewise, exploration of recovery practices was lacking across studies, despite being recognized as important for various fundamental functions among basketball practitioners (Pernigoni et al., 2022). In this way, survey data encompassing perceptions of end-users on the practices, efficacy, barriers, and facilitators of different injury prevention strategies (Bel et al., 2022) and recovery practices (Pernigoni et al., 2022) have been recently published and could help inform the development of future studies in these areas. Consequently, further research is encouraged exploring novel injury prevention and recovery strategies applicable to basketball players.
